# Naturally Bicarbonated Water Supplementation Does Not Improve Anaerobic Cycling Performance or Blood Gas Parameters in Active Men and Women

**DOI:** 10.3390/nu15245052

**Published:** 2023-12-09

**Authors:** Anthony M. Hagele, Johnathan L. Boring, Jessica M. Moon, Kyle L. Sunderland, Petey W. Mumford, Chad M. Kerksick

**Affiliations:** Exercise and Performance Nutrition Laboratory, College of Science, Technology, and Health, Lindenwood University, St. Charles, MO 63301, USA; ahagele@lindenwood.edu (A.M.H.); jessica.moon@ucf.edu (J.M.M.); ksunderland@lindenwood.edu (K.L.S.); pmumford@lindenwood.edu (P.W.M.)

**Keywords:** anaerobic exercise performance, blood gas kinetics, cycling, metabolic acidosis, blood lactate, ergogenic supplements, nutritional supplements

## Abstract

The completion of high-intensity exercise results in robust perturbations to physiologic homeostasis, challenging the body’s natural buffering systems to mitigate the accumulation of metabolic by-products. Supplementation with bicarbonate has previously been used to offset metabolic acidosis, leading to improvements in anaerobic exercise performance. Purpose: The purpose of this study was to investigate the presence of ergogenic properties in naturally occurring low-dose bicarbonated water and their effects on anaerobic cycling performance and blood gas kinetics in recreationally active men and women. Methods: Thirty-nine healthy, recreationally active men and women (28.1 ± 8.0 years, 169.8 ± 11.7 cm, 68.9 ± 10.8 kg, 20.1 ± 7.9% fat, $\dot{V}$O_2_peak: 42.8 ± 7.6 mL/kg/min) completed two separate testing sessions consisting of 15 cycling sprints (10 s sprint, 20 s active rest) against 7.5% of their body mass. Using a randomized, double-blind, placebo-controlled, parallel group study design, study participants consumed a 10 mL/kg dose of either spring water (SW) or bicarbonated mineral water (BMW) (delivering ~3 g/day of bicarbonate) for 7 days. Venous blood was collected before, immediately after, and 5 and 10 min after the sprint protocol and was analyzed for lactate and a series of blood gas components. After the completion of 15 cycling sprints, averages of peak and mean power for bouts 1–5, 6–10, and 11–15, along with total work for the entire cycling protocol, were calculated. All performance and blood gas parameters were analyzed using a mixed-factorial ANOVA. Results: pH was found to be significantly higher in the BMW group immediately after (7.17 ± 0.09 vs. 7.20 ± 0.11; *p* = 0.05) and 10 min post exercise (7.21 ± 0.11 vs. 7.24 ± 0.09; *p* = 0.04). A similar pattern of change was observed 5 min post exercise wherein pH levels in the SW group were lower than those observed in the BMW group; however, this difference did not achieve statistical significance (*p* = 0.09). A statistical trend (*p* = 0.06) was observed wherein lactate in the BMW group tended to be lower than in the SW group 5 min post exercise. No significant main effect for time (*p* > 0.05) or group × time interactions (*p* > 0.05) for the total work, average values of peak power, or average values of mean power were observed, indicating performance was unchanged. Conclusion: One week of consuming water with increased bicarbonate (10 mL/kg; ~3 g/day bicarbonate) showed no effect on anaerobic cycling performance. BMW decreased blood lactate concentrations 5 min after exercise and increased blood pH immediately and 10 min after exercise.

## 1. Introduction

The completion of high-intensity exercise results in robust perturbations to physiologic homeostasis, challenging the body’s natural buffering systems to mitigate the accumulation of metabolic by-products. Exercise-induced fatigue is attributed to several factors, primarily the depletion of energetic substrates and the accumulation of metabolic by-products [1,2]. When exercise intensity is low, the ATP demand of muscle contraction is met by mitochondrial respiration; however, as exercise intensity increases beyond what can be met by oxidative phosphorylation, ATP demand outpaces ATP production [3]. In scenarios with heightened energy output (such as repeated running or cycling sprints), a greater reliance is placed on the anaerobic glycolytic system, yielding higher concentrations of the metabolic by-products H^+^, inorganic phosphate, and others [3]. This elevation in metabolic by-product concentration is associated with impairments in the rate of anaerobic glycolysis, augmented fatigue, and performance decrements [4].

The primary means of the body to offset the accumulation of H^+^ ions and the concomitant decrease in intramuscular and venous pH is through the bicarbonate buffering system [5,6]. During intense physical effort, accumulation of H^+^ ions and carbon dioxide occurs as a consequence of greater metabolic demand. Bicarbonate (HCO_3_), an extracellular buffer synthesized by the kidneys, assumes a crucial role in sequestering the surplus of H^+^ ions produced in anaerobic glycolysis and CO_2_ as a metabolic by-product [7]. During intense exercise, CO_2_ is produced as a metabolic by-product and is bound to H_2_O, forming carbonic acid (H_2_CO_3_), and the bicarbonate present in the blood interacts with the accumulated H^+^ ions and ultimately dissociates into CO_2_ and H_2_O, with the subsequent CO_2_ released during exhalation. Muscle‘s capacity to buffer the increased H^+^ levels and maintain pH levels has been correlated with the extent of fatigue during intermittent sprint exercise [1].When the rate of H^+^ and lactate production exceeds its buffering capacity, a decline in performance indicators like power and total work have been observed [8].

Given the relationship between exercise-induced acidosis and fatigue, previous research has supported the efficacy of ingesting potential buffering agents such as bicarbonate to mitigate metabolic acidosis and improve anaerobic exercise performance [5,9,10,11]. Bicarbonate supplementation has been shown to be effective at improving buffering capacity during high-intensity exercise lasting 10–180 s in duration [12,13,14]. Matson et al. [15] reported improvements in exercise capacity in events like the 400–800 m run, while Lindh and colleagues [16] reported that bicarbonate improved 200 m freestyle swimming performance in elite male swimmers. Similarly, Marriott et al. [17] demonstrated a 23% improvement in running performance coupled with a reduction in perceived exertion in male team-sport athletes following sodium bicarbonate ingestion.

Efficacious supplementation with bicarbonate that is void of adverse events that complicate well-being and promote optimal physical performance continues to be an area of investigative focus. As such, research questions surrounding dosing amount, timing, and type (e.g., liquid, capsules, tablets, powder) have all been and continue to be explored surrounding bicarbonate. A dose of 0.3 g/kg most commonly appears in the literature and has the most consistent support for ergogenic outcomes (ISSN position stand). Most commonly, bicarbonate doses of 0.3 g/kg administered 60–90 min prior to exercise have shown ergogenic properties [18,19]. A critical consideration, however, remains the individual responses observed and the potential for gastrointestinal complications. Cameron et al. [20] noted a significant occurrence of gastrointestinal distress following an acute bicarbonate dose of 0.3 g/kg bodyweight 60 min prior to activity. The authors attributed the lack of performance improvement in part to the frequency and severity of gastrointestinal symptoms. In addition to the potential for gastrointestinal challenges, other authors have reported consistent variability when peak pH (used as a proxy for bicarbonate) levels are reached [21] and how varying the timing of ingestion may impact resulting performance [22]. Beyond these findings, bicarbonate ingestion is commonly achieved via dry powder or capsules, which may be a factor contributing to the consistently observed adverse outcomes [23]. To address some of these challenges, researchers have begun to explore the potential for lower doses, but some initial attempts have not been able to demonstrate the ergogenic properties observed more commonly at higher dosages [23,24].

A relatively new approach to bicarbonate administration is the ingestion of spring water samples from various parts of the world which have high amounts of bicarbonate and include varying compositions of minerals and electrolytes. These naturally sourced water options offer unique opportunities to consume a beverage that naturally contains bicarbonate in amounts that are severalfold higher than what is commonly found in standard spring water but is still a dosage that much lower than what has been used previously [23]. With the burgeoning popularity of hydration from global consumers in beverage form and powdered packets of dry ingredients, the interest in identifying the efficacy of a beverage that could offer hydration, vitamins, minerals, and added bicarbonate could attract significant consumer demand while also offering ergogenic efficacy. An initial study by Chycki et al. [25] explored the potential ergogenic properties of ingesting 3.2–3.4 L/day of water with naturally increased amounts of bicarbonate (4002.02 ± 120.3 mg/L) for three weeks and as part of rehydration protocol after inducing 3% body mass from exercise. These authors reported improved anaerobic performance in a 2 × 30 s upper and lower body Wingate test, increased lactate, and an improved rate of rehydration after consuming water with increased amounts of bicarbonate. Importantly, the participants in this study were elite judo athletes; however, much of the consumer interest in enhanced water and hydration products is from non-elite, recreationally active sporting individuals who would be less physically trained than the participants in the Chycki study. Considering the known physiological changes that occur in buffering potential with ongoing high-intensity physical training [26], the potential for a lower dose of bicarbonate mineral water ingestion to improve the performance of less-conditioned individuals remains to be determined.

For these reasons, the purpose of this study was to investigate the ergogenic effect of ingesting naturally occurring bicarbonate water at a dose of 10 mL/kg for seven days on performance variables, including peak power, average power, total work, blood gas kinetics, and ratings of perceived exertion following a bout of high-intensity cycling intervals. Our hypothesis is that regular consumption of naturally occurring bicarbonate water at a dose of 10 mL/kg (delivering ~3 g/day bicarbonate) for seven days will enhance anaerobic performance, lactate, and other blood gas parameters in recreationally trained men and women.

## 2. Materials and Methods

### 2.1. Overview of Research Design

The study was conducted using a randomized double-blind approach. Thirty-nine healthy, recreationally active men (*n* = 20) and women (*n* = 19) between the ages of 18 and 45 years of age were recruited to participate in this study. The study was conducted according to the guidelines of the Declaration of Helsinki and approved by the Institutional Review Board of Lindenwood University (IRB-20-112; approval date: 22 January 2020). Informed consent was obtained from all subjects involved in the study. Using G*Power 3.1 [27], a sample size of 15–20 participants per group would be needed if an effect size of 0.5–0.55 was realized with an alpha (α) level of 0.05 and an estimated power (1 − β) of 0.80. For each study visit, participants reported to the laboratory between 600 and 1000 h following an overnight fast (8–10 h). During the initial visit, height and weight were measured before fat-free mass and body fat were assessed using a bioelectrical impedance analysis (InBody 570, Beverly Hills, CA, USA). Participants were then positioned on a cycle ergometer (Lode Excalibur Sport, Groningen, The Netherlands), with the saddle and handlebar height and position recorded and standardized for each subsequent visit. Participants then completed a standardized warm-up consisting of 5 min of cycling at 50 W, 10 bodyweight squats, 20 walking lunges, 20 straight leg marches, and 20 walking quad stretches. Immediately following the warmup, participants performed a graded O_2_peak assessment on the cycle ergometer, beginning at 50 W and increasing one watt every two seconds (30 watts per minute) while maintaining a pedaling cadence between 60 and 100 rpm until volitional exhaustion. Following the $\dot{V}$O_2_peak assessment, participants completed two familiarization sessions of the repeated cycling sprint protocol. The first familiarization session occurred after a brief recovery (~15 min) from the $\dot{V}$O_2_peak assessment and consisted of an abbreviated repeated cycling sprint protocol (load set to 4.5% bodyweight). The second occurred during a subsequent visit in which participants completed the full repeated cycling sprint protocol (load set to 7.5% body mass). A minimum of 72 h separated the two familiarization sessions, and another 72 h was provided between the completion of the 2nd familiarization session and the onset of the supplementation protocol. Prior to beginning the supplementation protocol, participants completed pre-supplementation testing of the repeated cycling sprint protocol. Participants were then assigned in a randomized double-blind fashion in a matched-pair fashion according to body mass separately for each gender to ingest 10 mL/kg doses of either bicarbonated mineral water (BMW) (Borjormi, DS Borjormi, Tbilisi, Georgia) or a standard spring water (SW) placebo for seven days. Participants then returned to the laboratory to consume their final supplement beverage approximately 45–60 min before completing the post-supplementation repeated cycling sprint protocol.

### 2.2. Participants

Prior to participation, all recruited participants provided signed informed consent using an IRB-approved consent form. Thirty-nine healthy men and women (28.1 ± 8.0 years, 169.8 ± 11.7 cm, 68.9 ± 10.8 kg, 20.1 ± 7.9% fat, $\dot{V}$O_2_peak: 42.8 ± 7.6 mL/kg/min) successfully completed all aspects of the study protocol (Table 1). Inclusion criteria were age (18–45 years), being healthy and free of disease (as determined by the health history screening questionnaire), being physically active (reported at least two days of exercise per week for the past six months), and $\dot{V}$O_2_peak (>28 mL/kg/min; 8 METs). Any individual diagnosed with or being treated for cardiac, respiratory, circulatory, musculoskeletal, metabolic, immune, autoimmune, psychiatric, hematological, neurological, or endocrinological disorders or diseases was not allowed to participate. All study participants consuming any other supplementation apart from a multivitamin/mineral or protein supplementation were required to abstain from consuming any doses for 30 days prior to and during the study protocol. Study participants consuming a multivitamin/mineral or protein supplementation were instructed to maintain their current regimen throughout the study protocol.

### 2.3. Anthropometrics and Body Composition

Prior to all study visits, participants fasted for at least 8 h and abstained from exercise, caffeine, nicotine, and alcohol for at least 24 h. During the initial assessment, each participant’s height was measured to the nearest ± 0.5 cm using an analog wall-mounted stadiometer (HR-200, Tanita Corp, Inc., Tokyo, Japan) with their shoes removed and standing erect on flat feet. Body mass was measured prior to all study visits using a self-calibrating digital scale (Tanita BWB-627A, Tokyo, Japan) and was recorded to the nearest ±0.1 kg. To ensure any rapid changes in fluid masses due to any dietary or physical activity changes, participants whose body mass deviated by more than 2% throughout the study were excluded from participation. Fat mass and fat-free mass were determined using a bioelectrical impedance analyzer (InBody 570, Beverly Hills, CA, USA). Body composition analyses were conducted between 600 and 1000 h by trained research personnel. All assessments were completed according to device specifications. The reliability of the measurements obtained from the bioelectrical impedance analyzer was assessed in a cohort of healthy, college-aged males, revealing an intraclass correlation coefficient (ICC) of 0.992 for body fat %, 0.995 for fat mass, and 0.997 for fat-free mass.

### 2.4. Dietary Monitoring

Prior to their baseline testing, study participants completed a hand-written three-day food record (two weekdays and one weekend day). The three-day food log was provided to the participants for assistance when replicating their diets prior to subsequent testing sessions. In addition to the three-day hand-written food record, participants were instructed on how to complete an online dietary assessment tool (ASA-24; https://epi.grants.cancer.gov/asa24/; accessed on 7 December 2023) for the determination of baseline caloric and macronutrient intake. From this information, study participants were asked to replicate their dietary intake prior to each subsequent testing visit.

### 2.5. $\dot{V}$O_2_peak Assessment

During the first study visit, participants completed a peak oxygen consumption test ($\dot{V}$O_2_peak) using indirect calorimetry on a ParvoMedics TrueOne (Sandy, UT, USA) metabolic cart interfaced to a motorized cycle ergometer (Lode Excalibur Sport, Groningen, The Netherlands). The assessment utilized a ramp protocol, commencing with 30 s of unloaded baseline pedaling, followed by a progressive increase in power output of 30 W/min, beginning at 50 W, while maintaining a pedaling cadence between 60 and 100 rpm. The test was terminated when pedaling cadence fell more than 10 rpm below the participant’s self-selected cadence for more than 10 s despite strong verbal encouragement. Acceptance criteria for each $\dot{V}$O_2_peak assessment included the fulfillment of at least two of the three following criteria: (1) the achievement of a respiratory exchange ratio > 1.05, (2) a recorded heart rate within 10 bpm of the age-predicted maximal heart rate (HRmax = 208 − [0.7 × age]), and (3) $\dot{V}$O_2_ variation < 250 mL/min for two consecutive sampling points. Throughout the test, pulmonary gas exchange was determined as the highest $\dot{V}$O_2_peak over a 30 s period.

### 2.6. Repeated Cycling Sprints

Prior to beginning supplementation, and after 7 days of supplementation, all study participants completed a sprint cycling protocol consisting of 15 separate 10 s sprints on a Lode Excalibur Sport cycle ergometer (Lode Excalibur Sport, The Netherlands). A 20 s active rest period was provided after each 10 s effort. Consequently, the entire sprint bout lasted 450 s (7.5 min). The participants remained strapped into the bike pedals during the entire sprint protocol and were instructed to pedal at a low, self-selected-frequency pedaling speed each 20 s rest period. Prior to the onset of each 10 s sprint, a 5 s countdown was provided wherein participants were instructed to achieve their highest cycling cadence when resistance was applied. Participants were instructed to maintain their cadence as high as possible during each sprint, and strong verbal encouragement was provided throughout each sprint. Participants were not informed of the elapsed time of the sprint to prevent pacing and to ensure maximal effort. From each repeated cycling sprint protocol, peak power, mean power, and total work were calculated across the entire bout (all 15 sprints), in addition to the averages of these variables being calculated for sprints 1–5, 6–10, and 11–15.

### 2.7. Rating of Perceived Exertion and Visual Analog Scale

A rating of perceived exertion (RPE) and a fatigue visual analog scale (VAS) were recorded pre-exercise and following the completion of the 3rd, 6th, 9th, 12th, and 15th sprints. The RPE was assessed on a scale of 6–20, with 6 indicating the lowest level of exertion and 20 indicating the highest level of exertion. The VAS scale was completed using a 100 mm line anchored by “Lowest Possible Fatigue” and “Highest Possible Fatigue” to assess subjective ratings of fatigue.

### 2.8. Supplementation Protocol

In a randomized, double-blind, placebo-controlled fashion, participants supplemented at a dose of 10 mL/kg body mass for seven consecutive days with either standard spring water (as a placebo) or water with increased amounts of bicarbonate and other minerals ((BMW) Borjomi, IDS Borjormi, Tbilisi, Georgia). The dose of 10 mL/kg was decided upon through consultation with the sponsor as a dose that many recreational fitness enthusiasts may commonly consume each day. The mineral composition of the bicarbonate mineral water was as follows: bicarbonate (3700–4900 mg/L, chloride (230–480 mg/L, calcium (13–135 mg/L, magnesium (14–41 mg/L), sodium (940–1900 mg/L, potassium (11–42 mg/L), and other minerals (3.8–7.8 mg/L). Individuals assigned to complete the placebo condition were assigned to ingest bottled spring water with negligible bicarbonate. The composition of the spring water was also evaluated: bicarbonate (3.41–3.87 mg/L, chloride (0.33–0.52 mg/L, calcium (1.13–1.37 mg/L, magnesium (0.29–0.55 mg/L), sodium (0.96–1.49 mg/L, potassium (0.18–0.46 mg/L), and other minerals (0.41–0.96 mg/L). All bottles were identical in size and color with similar labeling. All supplements were stored in identical environmental conditions. Participants were instructed to ingest their doses at similar times of day. On testing days, participants ingested their required daily dose approximately 45–60 min prior to beginning their exercise tests. Participants completed a log to record their compliance with the supplementation protocol and any adverse reactions to their assigned supplement group. All randomization and blinding were completed by the manufacturer prior to shipment. To evaluate the effectiveness of the blinding protocol, participants reported which supplement condition they thought was administered to them following their final study visit.

### 2.9. Blood Lactate and Blood Gas Measurements

For each participant, venous blood samples were collected at study visits before and after the completion of the supplementation protocol to evaluate changes in blood lactate and blood gas parameters prior to, immediately after, and 5 min and 10 min after the completion of the sprint cycling protocol. For all blood samples, a small amount of blood (<1 mL) was collected from an IV catheter via a syringe and inserted into a 100 µL capillary tube. Participants were instructed to lie prone immediately following the sprint cycling protocol while blood was collected and until the final 10 min post sample was collected. All collected samples were analyzed using a portable blood gas analyzer (epoc Blood Analysis System, Epocal, Inc., Ottawa, ON, Canada) for sodium, potassium, chloride, anion gap, calcium, glucose, urea, creatinine, lactate, hematocrit, hemoglobin, pH, pCO_2_, pO_2_, TCO_2_, HCO_3_, base excess, and O_2_ saturation. The epoc blood gas analyzer has previously demonstrated high precision and strong correlation with other blood gas analyzers for all measured analytes [28].

### 2.10. Statistical Analysis

All analyses were completed using Microsoft Excel (v2307) and the Statistical Package for the Social Sciences (v23; SPSS Inc., Chicago, IL, USA). For all dependent measures, descriptive statistics (means and standard deviations) were calculated. Data were first analyzed for normality, skewness, and kurtosis. All non-normal data were log-transformed prior to analysis. For all statistical tests, data were considered statistically significant when the probability of type I error was 0.05 or less. The primary endpoints for this analysis include the peak power, mean power, and total work completed throughout the sprint cycling protocol, while secondary outcomes were the RPE, fatigue VAS, lactate, and other blood gas parameters. Three-way (visit × group × sprint) mixed-factorial ANOVAs were first performed on peak power, mean power, and total work to rule out any confounding influence of performance differences prior to supplementation. If no three-way interaction was observed, two-way mixed factorial ANOVAs (group × sprint) were computed using just the post-supplementation data. A similar approach was used for the RPE, fatigue VAS, lactate, and all blood gas parameters wherein separate 2 × 2 mixed-factorial ANOVAs were completed first using all pre-supplementation values to evaluate if any between-group differences were evidenced prior to supplementation. If no significant group × time interactions were observed, then only the post-supplementation values were used for all subsequent statistical analyses and are reported throughout this paper. In all instances, when a significant group, treatment and/or interaction alpha was observed, Least Significant Difference (LSD) post hoc analyses were performed to determine where significance was obtained. When the sphericity assumption was violated, the Huynh–Feldt correction was used for all statistical determination when eta exceeded 0.75, and the Greenhouse–Geiser correction was used when eta was less than 0.60. Area under the curve values were computed using Microsoft Excel for the total work completed during all sprints.

## 3. Results

### 3.1. Participant Demographics

**Figure 1:**
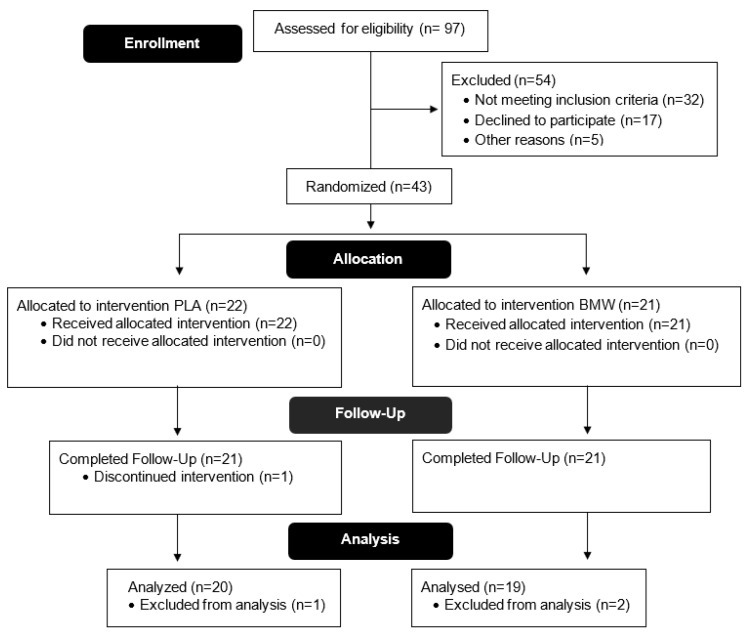
CONSORT (Consolidated Standards of Reporting Trials) diagram.

Thirty-nine healthy men (*n* = 20) and women (*n* = 19) (28.1 ± 8.0 years, 169.8 ± 11.7 cm, 68.9 ± 10.8 kg, 20.1 ± 7.9% fat, $\dot{V}$O_2_peak: 42.8 ± 7.6 mL/kg/min) completed all aspects of the study protocol (See Table 1). There were no significant differences in participant demographics between supplement conditions (*p* > 0.05). As seen in Figure 1, 97 people were assessed for eligibility, of which 54 were excluded and 43 were randomized into the protocol. Subsequently, one participant discontinued the intervention, and two participants were excluded from the final analysis due to noncompliance with the protocol. In consideration of our blinding, 76.2% of participants successfully determined which supplementation they were assigned, while 23.8% of participants were not able to properly identify their assigned group.

### 3.2. Dietary Intake

Of the 39 people who completed all aspects of the study protocol, only 35 provided suitable food records (See Table 2). Within each supplementation group, no changes (*p* > 0.05) were observed in dietary intake parameters assessed throughout the study protocol. Further, no statistically significant group × time interactions were observed.

### 3.3. Total Work

**Figure 2:**
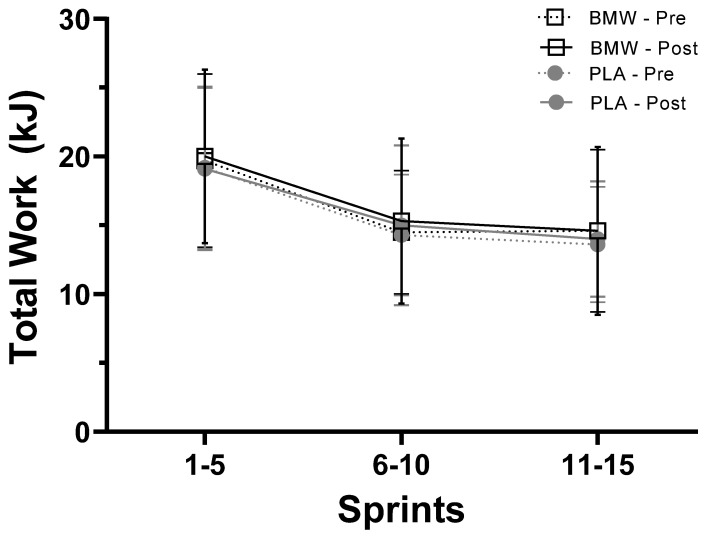
Total work (kJ) produced during sprints 1–5, 6–10, and 11–15 (mean ± SD) for BMW (open squares and black) and placebo (closed circles and gray) groups. Pre-supplementation = dotted line; post-supplementation = solid line.

Total work was computed for each sprint and summed across all 15 sprints prior to data analysis. No three-way interaction was observed (*p* = 0.150) for total work production. From there, changes in total work were evaluated using separate 2 × 2 mixed-factorial ANOVAs across the entire sprint cycling bout (sprints 1–15) and separately for sprints 1–5, 6–10, and 11–15. As seen in Figure 2, no significant main effect of time (*p* = 0.17) or group × time interaction (*p* = 0.87) was observed when the total work completed across all sprints was calculated. Similar outcomes were observed with separate mixed-factorial ANOVAs for total work following sprints 1–5 (time, *p* = 0.76; group × time, *p* = 0.31), sprints 6–10 (time, *p* = 0.11; group × time, *p* = 0.79), and sprints 11–15 (time, *p* = 0.14; group × time, *p* = 0.32).

### 3.4. Average Power

**Figure 3:**
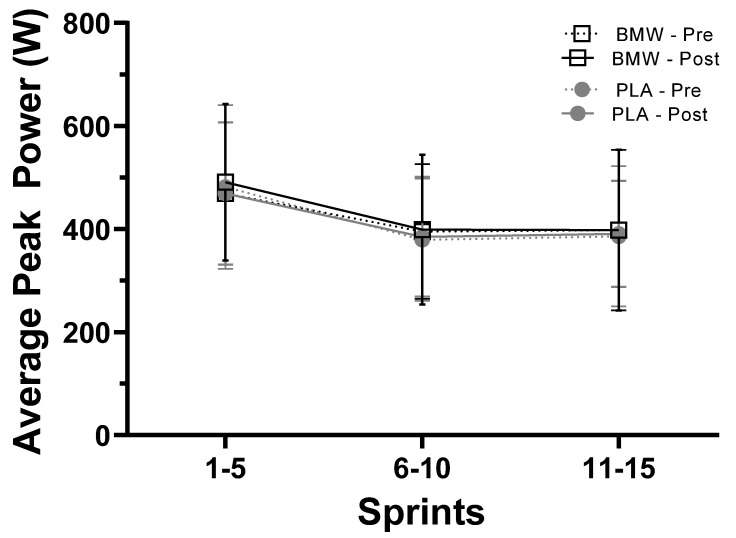
Average peak power (W) produced during sprints 1–5, 6–10, and 11–15 (mean ± SD) for BMW (open squares and black) and placebo (closed circles and gray) groups. Pre-supplementation = dotted line; post-supplementation = solid line.

Software (Lode Ergometry Manager, v10) on the cycle ergometer calculated the average power generated across each 10 s sprint. An average of each mean power was computed for all sprints (1–15) and sprints 1–5, 6–10, and 11–15. No three-way interaction was observed (*p* = 0.152) for mean power production. From there, changes in mean power production were evaluated using separate 2 × 2 mixed-factorial ANOVAs across the entire sprint cycling bout (sprints 1–15) and for sprints 1–5, 6–10, and 11–15. As seen in Figure 3, the changes observed in mean power for all sprints (1–15) indicated no significant main effect of time (*p* = 0.17) or group × time interaction (*p* = 0.88). Similar outcomes were observed with separate mixed-factorial ANOVAs for the mean power achieved after sprints 1–5 (time, *p* = 0.76; group × time, *p* = 0.32), sprints 6–10 (time, *p* = 0.13; group × time, *p* = 0.78), and sprints 11–15 (time, *p* = 0.14; group × time, *p* = 0.31).

### 3.5. Average Peak Power

**Figure 4:**
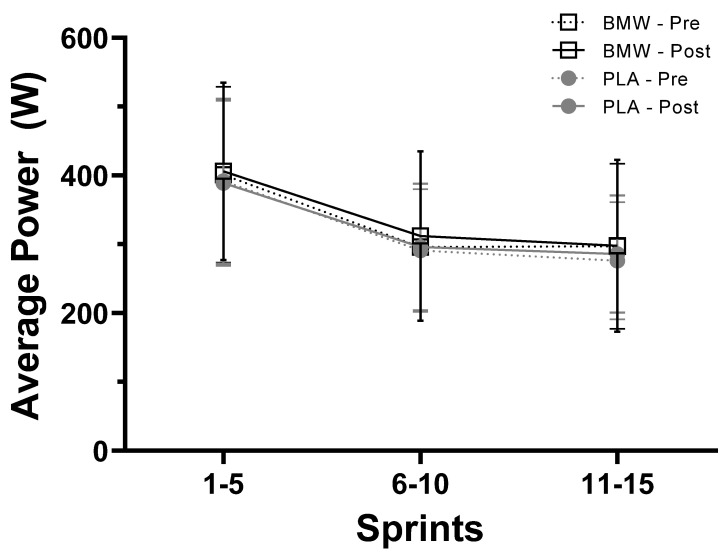
Average power (W) produced during the duration of sprints 1–5, 6–10, and 11–15 (mean ± SD) for BMW (open squares and black) and placebo (closed circles and gray) groups. Pre-supplementation = dotted line; post-supplementation = solid line.

Peak power was also computed after each 10 s sprint. An average of the peak powers generated was computed for sprints (1–15) and sprints 1–5, 6–10, and 11–15. No three-way interaction was observed (*p* = 0.374) for peak power production. From there, changes in peak power production were evaluated using separate 2 × 2 mixed-factorial ANOVAs across the entire sprint cycling bout (sprints 1–15) and for sprints 1–5, 6–10, and 11–15. As seen in Figure 4, the changes observed in peak power for all sprints (1–15) indicated no significant main effect of time (*p* = 0.45) or group × time interaction (*p* = 0.56). Similar outcomes were observed with separate mixed-factorial ANOVAs for the mean power achieved after sprints 1–5 (time, *p* = 0.44; group × time, *p* = 0.27), sprints 6–10 (time, *p* = 0.33; group × time, *p* = 0.87), and sprints 11–15 (time, *p* = 0.05; group × time, *p* = 0.89).

### 3.6. Rating of Perceived Exertion and Visual Analog Scales of Fatigue

No significant group × time interaction was observed for the RPE (*p* = 0.88) prior to to supplementation. To evaluate changes in the RPE after supplementation, a 2 × 6 (group [SW vs. BMW] × sprint) mixed-factorial ANOVA with repeated measures on sprint was completed. RPE values collected after the completion of the supplementation protocol exhibited a significant main effect of time (*p* < 0.001), while no significant group × time interaction (*p* = 0.99) was observed (Table 3).

Fatigue VAS values exhibited no significant group × time interaction (*p* = 0.53) prior to to supplementation. To evaluate changes in fatigue VAS after supplementation, a 2 × 6 (group [SW vs. BMW] × sprint) mixed-factorial ANOVA with repeated measures on sprint was completed. Fatigue VAS values collected after the completion of the supplementation protocol exhibited a significant main effect of time (*p* < 0.001), while no significant group × time interaction (*p* = 0.75) was observed (Table 3).

### 3.7. Lactate, pH, and Other Blood Gas Parameters

Lactate changes prior to supplementation indicated no group × time interaction for lactate (*p* = 0.23). After supplementation, a significant main effect of time (*p* < 0.001) was observed, while no group × time interaction was determined (*p* = 0.17). Additionally, no significant group × time interaction (*p* = 0.98) was observed using area under the curve calculations for lactate. pH changes prior to supplementation indicated no group × time interaction for pH (*p* = 0.32). After supplementation, a significant main effect of time (*p* < 0.001) was observed, while no group × time interaction was determined (*p* = 0.85). pCO_2_ changes prior to supplementation indicated no group × time interaction for pCO_2_ (*p* = 0.45). After supplementation, a significant main effect of time (*p* < 0.001) was observed, while no group × time interaction was determined (*p* = 0.55). pO_2_ changes prior to supplementation indicated no group × time interaction for pO_2_ (*p* = 0.14). After supplementation, a significant main effect of time (*p* < 0.001) was observed, while the group × time interaction tended to be different (*p* = 0.06). HCO_3_ changes prior to supplementation indicated no group × time interaction for HCO_3_ (*p* = 0.51). After supplementation, a significant main effect of time (*p* < 0.001) was observed, while no group × time interaction was determined (*p* = 0.18). CO_2_ changes prior to supplementation indicated no group × time interaction (*p* = 0.23). After supplementation, a significant main effect of time (*p* < 0.001) was observed, while no group × time interaction was determined (*p* = 0.18). Base excess (ECF) changes prior to supplementation indicated no group × time interaction (*p* = 0.64). After supplementation, a significant main effect of time (*p* < 0.001) was observed, while no group × time interaction was determined (*p* = 0.26). Base excess (B) changes prior to supplementation indicated no group × time interaction (*p* = 0.70). After supplementation, a significant main effect of time (*p* < 0.001) was observed, while no group × time interaction was determined (*p* = 0.32).

## 4. Discussion

The main objective of the present study was to evaluate the effects of seven days of BMW supplementation on selected anaerobic performance variables in recreationally trained men and women. The key findings of the present study were that seven days of BMW ingestion exerted no improvements in any measure of exercise performance as measured in the present study. Along these lines, BMW ingestion led to unfavorable increases in blood pH levels immediately, five minutes, and ten minutes after exercise. Finally, BMW ingestion significantly decreased blood lactate concentrations five minutes after completing an intermittent, high-intensity bout of cycling sprints.

Results from the present study differ from previous investigations involving bicarbonate supplementation; several studies have reported significant improvements in anaerobic performance following bicarbonate supplementation [29,30,31,32]. In this respect, Grgic and colleagues [23], in a position stand published by the International Society of Sports Nutrition, concluded that doses of bicarbonate ranging from 0.2 to 0.5 g/kg body mass could improve performance in high-intensity muscular endurance activities spanning 30 s to 12 min. These authors and others have highlighted that a key consideration when examining the potential efficacy of bicarbonate supplementation is to ensure the activation and heavy involvement of the glycolytic energy system under anaerobic conditions [14]. Several indicators exist from our findings that heavy activation of anaerobic metabolism occurred throughout our sprint cycling protocol. For example, peak and mean power as well as total work (Figure 2, Figure 3 and Figure 4) produced from the beginning to the end of the sprint protocol saw sharp and robust reductions. Furthermore, venous lactate concentrations increased from resting values that ranged from 1.5 to 2.05 mM to values that ranged from 13.8 to 15.7 mM five minutes after the completion of the sprint protocol (Table 4). Similarly, sharp, and drastic reductions in pH, pCO_2_, and HCO_3_ were also observed (Table 4), which provide additional evidence that the sprint protocol utilized did sufficiently challenge the participants’ anaerobic energy systems.

While bicarbonate ingestion appears to exert performance improvements for single-bout exercise, its ergogenic potential appears to be more pronounced in multiple bouts of maximal exercise, likely due to the increased acidosis and the drastic perturbations to homeostasis that occur with subsequent exercise bouts [23]. To this point, Costill et al. [33], used a 4 × 1 min cycling protocol with a fifth sprint effort to exhaustion. In this model, bicarbonate ingestion (0.2 g/kg) was responsible for an average increase in exhaustion time of 47 s. Similarly, Artioli et al. [34] demonstrated significant increases in peak and mean power in bouts 3 and 4 during a 4 × 30 s arm-cranking test with the consumption of bicarbonate (0.3 g/kg). Ergogenic outcomes were also reported by Price et al. [35], who had eight healthy male subjects complete a 30 min bout of intermittent cycling exercise that changed in intensity throughout the protocol. When bicarbonate was provided (0.3 g/kg) prior to the exercise bout, significant improvements in cycling performance were identified. Alternatively, studies are available that also reported on the inability of bicarbonate to yield improvements in performance. Briefly, Joyce and colleagues [36] failed to identify improvements in 200 m swim performance times after 1–4 days of supplementing with bicarbonate (0.3 g/kg) in highly trained male swimmers. Likewise, Vanhatalo et al. [37] showed no improvements in 3 min all-out cycling among active males consuming bicarbonate (0.3 g/kg) 60 min prior to exercise. Similarly, Zabala et al. [38] showed no significant improvements in 3 × 30 s Wingate performance with bicarbonate (0.3 g/kg) in elite male BMX riders. In recent years, the individualization of bicarbonate dosing [39,40] and the timing of bicarbonate administration [22] have been examined as key factors that may impact the potential for bicarbonate to impact performance.

Several limitations are worthy of discussion that may have impacted our outcomes. First and most importantly, the dose of bicarbonate (~3 g/d) utilized in the BMW was lower than the doses most commonly reported to show an ergogenic outcome. The current consensus suggests that achieving ergogenic effects with bicarbonate supplementation requires a dose of 0.3 g/kg body mass [13,41]. Nonetheless, previous investigations have explored deviations from this standard dosage to better comprehend the optimal dosage required. Ferreira et al. [42] used a single 0.1 g/kg dose in male cyclists who performed a cycling test to exhaustion and demonstrated no ergogenic effects when compared to a placebo. Similarly, Horswill et al. [43] used three separate single doses of 0.1, 0.15, and 0.2 g/kg of sodium bicarbonate prior to testing cycling sprint performance in endurance-trained cyclists (4 × 2 min intervals) and observed no performance improvements for either dose. Others have shown null outcomes when using a dose between 0.1 and 0.15 g/kg, suggesting the minimal effective dose appears to be around 0.2 g/kg [23,33,44], while others have demonstrated performance enhancement at 0.2 and 0.3 g/kg doses [40,45]. Alternatively, Chycki et al. [25] reported significant improvements in anaerobic performance and elevations in blood lactate following 2 × 30 s upper- and lower-limb Wingates among elite Judo athletes using a non-randomized, single-blind study design following a three-week dosing regimen of bicarbonate-rich water (3.2–3.4 L/day) and acute rehydration of 150% of lost body mass after achieving 3% body mass loss during a dehydration protocol. While the compositions of the water consumed during the Chycki study [25] and the present study were similar, a combination of other factors could have impacted our outcomes, including discrepancies in the training status of participants (elite male judo athletes vs. recreational males and females); a longer duration of low-dose supplementation (7 days vs. 21 days), the amount of fluid (and subsequently, the amount of bicarbonate) consumed each day (10 mL/kg vs. 3.2–3.4 L/day), and the rapid acute ingestion of bicarbonate water (150% of lost body mass) after a 3% loss in body mass with the bicarbonated water.

Furthermore, and as mentioned previously, our investigation employed a multi-day dosing protocol for bicarbonate supplementation. While the majority of studies employed an acute bicarbonate ingestion protocol [11,19,22,33,34,42,43,46,47,48,49], a subset of investigations extended supplementation over several consecutive days, typically 3 to 7 days, prior to assessing exercise performance [30,50,51]. McNaughton et al. [51] administered a daily dose of 0.5 g/kg, divided into four smaller doses ingested throughout the day, over the course of five days preceding testing. This regimen yielded favorable improvements in total work and peak power during a 60 s cycling exercise test. Conversely, other investigations adopting a multi-day dosing strategy did not yield significant ergogenic outcomes with a 0.3 g/kg per day dose [36,52]. It is noteworthy that Grgic and colleagues [23] of the International Society of Sports Nutrition position stand proposed the possibility that larger doses of 0.4 to 0.5 g/kg per day may be required to elicit performance improvements with multi-day dosing regimens. Thus, while our supplementation regimen did persist for several more days than is typically employed, the lower total dose of bicarbonate was likely a key consideration for our inability to demonstrate an ergogenic outcome and the possibility that the timing and pattern of ingestion may have also been contributors to our null outcomes [39,40].

Another primary consideration for our findings relates to the training status of our recruited cohort. Many studies involving bicarbonate supplementation recruited highly trained, elite competitors, while we selectively recruited recreationally active men and women. In consideration of this, it is possible that the intensity of the exercise employed in our study overwhelmed the body’s buffering systems, potentially attenuating the supplement’s capacity to exert a performance-enhancing influence (not withstanding any shortcomings brought forth with our supplementation protocol). To this point, the exercise bout resulted in an exceptional increase in fatigue and the activation of the anaerobic exercise systems, underscoring the effectiveness of our chosen exercise protocol at inducing metabolic acidosis. Our findings align with earlier evidence from several studies involving recreationally trained populations which likewise demonstrated no discernible improvements in performance. For instance, Brisola et al. [46] observed no improvements in time to exhaustion following 0.3 g/kg bicarbonate supplementation administered 90 min before exercise among moderately active males who were subjected to high-intensity running at 110% $\dot{V}$O_2_max. Similarly, Siegler et al. [22] demonstrated no improvements in speed, power, or total running distance, as well as no difference in blood buffering capacity, in recreationally active males following an exercise test consisting of 10 sets of 10 s sprints with bicarbonate ingestion of 0.3 g/kg at 60, 120, or 180 min prior to exercise. In contrast with these findings, others have observed ergogenic effects of bicarbonate supplementation in both recreationally active males and females. Deb et al. [48] conducted a randomized, double-blind crossover study involving recreationally active males who engaged in repeated 60 s cycling sprints with a 20 s recovery between bouts until exhaustion with 0.3 g/kg bicarbonate supplementation compared to a placebo. Their results revealed improvements in total work completed and exercise tolerance. In a similar manner, Gough et al. [53], using a randomized crossover design, demonstrated increased time to fatigue following two separate exhaustive cycling trials at 100% peak power separated by a 90 min passive recovery period in active males with a 0.3 g/kg bicarbonate dose administered after the first cycling trial. Further reinforcing these findings, using a double-blind crossover design, McNaughton et al. [49] exhibited enhancements in total work and peak power during 60 s of maximal cycling among moderately trained females ingesting a bicarbonate dose of 0.3 g/kg 90 min prior to exercise.

Changes in dietary intake or poor compliance can safely be ruled out as confounding factors as there were no significant differences in energy or macronutrient intake during the day prior to the pre-supplementation testing period during the supplementation testing period, and compliance with the supplementation protocol was excellent, as observed in the self-reported supplement diaries of the participants. Moreover, two familiarization trials of the repeated sprint exercise were implemented to help reduce the potential impact of a learning effect for the exercise protocol. Finally, the effectiveness of the supplement condition blinding from participants may be questioned as nearly 75% of the participants were able to correctly identify whether they were on placebo or bicarbonate water when asked which supplemental condition they thought they were assigned. This was likely due to the taste and texture of the bicarbonate water as it offered a more “mineralated” mouth feel when compared to standard water. The extent to which this impacted how hard a participant did or did not strive to complete the post-testing session remains unknown; however previous scientific reviews have highlighted the power of the placebo effect [54,55]. Researchers should take notice of this as a distinct advantage of using capsulated bicarbonate versus other modes of administration.

## 5. Conclusions

In conclusion, the results of the present investigation indicate that seven days of consuming mineral water with increased amounts bicarbonate at a dose of 10 mL/kg (delivering approximately 3 g of bicarbonate per day) exerts limited to no potential to augment exercise performance, lactate, pH, and other blood gas parameters in recreationally active men and women. Future research on liquid formulations should explore higher doses of bicarbonate (if palatable) or more aggressive hydration regimens to better understand its impact on anaerobic performance and the potential hydration benefits of bicarbonate-rich mineral water; alternatively, an additional recommendation would be to employ a more individualized supplementation approach, as reported by Gough et al. [40], who reported similar performance enhancements with higher (0.3 g HCO_3_/kg) and lower (0.2 g HCO_3_/kg) doses.

## Figures and Tables

**Table 1:** Baseline age, height (cm), weight (kg), bodyfat percentage, $\dot{V}$O_2_max (mL O_2_/kg/min), and metabolic equivalents (METs).

	Condition	Mean ± SD	Minimum	Maximum
Age (years)	SW	28.7 ± 9.1	20	45
	BMW	27.5 ± 7.0	20	44
Height (cm)	SW	170.8 ± 7.9	157	185
	BMW	168.9 ± 14.4	122.5	189
Weight (kg)	SW	69.8 ± 12.5	46.5	94.5
	BMW	68.2 ± 9.2	49.8	89
Fat Percent (%)	SW	20.5 ± 8.5	7	35.7
	BMW	19.7 ± 7.3	10	37
$\dot{V}$O_2_max (mL O_2_/kg/min)	SW	42.1 ± 8.1	29.4	56.1
	BMW	43.3 ± 7.3	28.7	57.2
METs	SW	12.0 ± 2.3	8.4	16
	BMW	12.4 ± 2.1	8.2	16.4

SW = spring water; BMW = bicarbonated mineral water; cm = centimeters; kg = kilograms; % = percent; mL = milliliter; METs = metabolic equivalents; SD = standard deviation.

**Table 2:** Dietary intake.

Variable		Pre Supplementation	Post Supplementation	Between Group (*p*)	
Energy (kcal/day)	SW	2287 ± 729	2189 ± 714	Time	0.547
	BMW	2135 ± 887	2096 ± 1084	G × T	0.796
Protein (g/day)	SW	125.1 ± 52.3	102.6 ± 35.9	Time	0.114
	BMW	104.2 ± 44.9	106.1 ± 58.6	G × T	0.065
Carbohydrates (g/day)	SW	232.7 ± 94.8	251.8 ± 104.4	Time	0.626
	BMW	230.1 ± 135.2	226.6 ± 155.6	G × T	0.455
Fat (g/day)	SW	97.0 ± 35.2	86.7 ± 38.5	Time	0.216
	BMW	91.0 ± 41.2	86.2 ± 40.1	G × T	0.645
Potassium (mg/day)	SW	2909 ± 1456	2977 ± 1340	Time	0.272
	BMW	2808 ± 1328	3025 ± 1692	G × T	0.563
Sodium (mg/day)	SW	4614 ± 1523	4140 ± 1366	Time	0.415
	BMW	3787 ± 1405	3837 ± 2096	G × T	0.316
Calcium (mg/day)	SW	1246 ± 896	1097 ± 742	Time	0.989
	BMW	1023 ± 628	1175 ± 776	G × T	0.141

SW = spring water; BMW = bicarbonated mineral water; g = grams; mg = milligrams. Pre-Supplementation = before onset of supplementation protocol. Post-Supplementation = after completion of supplementation protocol. G × T = interaction between group and time.

**Table 3:** Post-supplementation RPE and fatigue VAS.

	Start	Sprint 3	Sprint 6	Sprint 9	Sprint 12	Sprint 15	Between Group (*p*)	
RPE								
SW	6.2 ± 0.7	11.2 ± 2.2	14.1 ± 2.2	16.0 ± 2.4	17.3 ± 2.3	18.6 ± 2.0	Time	<0.001
BMW	6.6 ± 1.2	11.4 ± 2.1	14.4 ± 2.0	16.5 ± 1.8	17.9 ± 1.7	18.9 ± 1.6	G × T	0.99
Fatigue VAS								
SW	4.3 ± 6.1	20.6 ± 13.2	49.5 ± 16.2	68.4 ± 17.8	80.2 ± 13.3	91.1 ± 18.7	Time	<0.001
BMW	4.5 ± 4.9	28.7 ± 15.4	53.1 ± 15.9	69.5 ± 15.2	81.8 ± 13.5	88.4 ± 12.2	G × T	0.75

RPE = rating of perceived exertion; VAS = visual analog scale; SW = spring water; BMW = bicarbonated mineral water. Time = main effect of time; G × T = interaction between group and time.

**Table 4:** Lactate, pH, and other blood gas parameters.

	Pre	Immediate Post	5 Min Post	10 Min Post	Between Group (*p*)	
Lactate (mM)						
SW	2.1 ± 0.9	12.5 ± 3.1	13.8 ± 3.7	13.0 ± 4.2	Time	<0.001
BMW	1.9 ± 0.9	12.5 ± 5.5	15.7 ± 4.4	15.0 ± 4.5	G × T	0.17
pH						
SW	7.3 ± 0.	7.17 ± 0.1	7.19 ± 0.1	7.21 ± 0.1	Time	<0.001
BMW	7.3 ± 0.	7.20 ± 0.1	7.20 ± 0.1	7.24 ± 0.1	G × T	0.85
PCO_2_ (mmHg)						
SW	56.8 ± 6.6	60.3 ± 13.2	44.9 ± 9.0	41.6 ± 7.0	Time	<0.001
BMW	55.0 ± 5.7	58.8 ± 12.1	39.8 ± 5.2	38.3 ± 5.0	G × T	0.55
PO_2_ (mmHg)						
SW	22.5 ± 5.6	27.7 ± 13.3	49.3 ± 10.3	50.6 ± 15.6	Time	<0.001
BMW	25.1 ± 6.1	24.6 ± 13.6	58.6 ± 16.2	57.5 ± 15.6	G × T	0.06
HCO_3_ (mM)						
SW	28.7 ± 2.2	21.9 ± 3.2	16.9 ± 3.2	16.8 ± 3.1	Time	<0.001
BMW	29.8 ± 2.0	23.1 ± 4.6	15.9 ± 3.8	16.6 ± 4.1	G × T	0.18
CO_2_ (mM)						
SW	30.5 ± 2.4	23.7 ± 3.4	18.3 ± 3.3	18.1 ± 3.1	Time	<0.001
BMW	31.5 ± 2.1	24.9 ± 4.6	17.2 ± 3.9	17.8 ± 4.2	G × T	0.18
Base Excess (ECF)						
SW	2.5 ± 2.1	−6.6 ± 3.6	−11.3 ± 4.0	−11.0 ± 4.3	Time	<0.001
BMW	4.1 ± 1.9	−4.9 ± 6.0	−12.1 ± 5.0	−10.8 ± 5.4	G × T	0.26
Base Excess (B)						
SW	1.0 ± 1.5	−7.7 ± 3.3	−11.2 ± 3. 8	−10.7 ± 4.3	Time	<0.001
BMW	4.1 ± 1.9	−4.9 ± 6.0	−12.1 ± 5.0	−10.8 ± 5.4	G × T	0.26

SW = spring water; BMW = bicarbonated mineral water. Pre = before onset of supplementation protocol. Post = after completion of supplementation protocol. G × T = interaction between group and time. PCO_2_ = partial pressure of carbon dioxide. PO_2_ = partial pressure of oxygen. HCO_3_ = bicarbonate concentration. CO_2_ = carbon dioxide. ECF = extracellular fluid. B = blood. mM = millimoles per liter.

## Data Availability

The datasets used and/or analyzed during the current study are available from the corresponding author upon reasonable request.
